# Measuring the Population Burden of Injuries—Implications for Global and National Estimates: A Multi-centre Prospective UK Longitudinal Study

**DOI:** 10.1371/journal.pmed.1001140

**Published:** 2011-12-06

**Authors:** Ronan A. Lyons, Denise Kendrick, Elizabeth M. Towner, Nicola Christie, Steven Macey, Carol Coupland, Belinda J. Gabbe, on behalf of the UK Burden of Injuries Study Group

**Affiliations:** 1 Centre for Health Information Research and Evaluation, College of Medicine, Swansea University, United Kingdom; 2 Division of Primary Care, University of Nottingham, Nottingham, United Kingdom; 3 Centre for Child and Adolescent Health, University of the West of England, Oakfield House, Bristol, United Kingdom; 4 Centre for Transport Studies, University College London, London, United Kingdom; 5 School of Public Health and Preventive Medicine, Monash University, Victoria, Australia; Research Center INSERM U897, France

## Abstract

**Background:**

Current methods of measuring the population burden of injuries rely on many assumptions and limited data available to the global burden of diseases (GBD) studies. The aim of this study was to compare the population burden of injuries using different approaches from the UK Burden of Injury (UKBOI) and GBD studies.

**Methods and Findings:**

The UKBOI was a prospective cohort of 1,517 injured individuals that collected patient-reported outcomes. Extrapolated outcome data were combined with multiple sources of morbidity and mortality data to derive population metrics of the burden of injury in the UK. Participants were injured patients recruited from hospitals in four UK cities and towns: Swansea, Nottingham, Bristol, and Guildford, between September 2005 and April 2007. Patient-reported changes in quality of life using the EQ-5D at baseline, 1, 4, and 12 months after injury provided disability weights used to calculate the years lived with disability (YLDs) component of disability adjusted life years (DALYs). DALYs were calculated for the UK and extrapolated to global estimates using both UKBOI and GBD disability weights. Estimated numbers (and rates per 100,000) for UK population extrapolations were 750,999 (1,240) for hospital admissions, 7,982,947 (13,339) for emergency department (ED) attendances, and 22,185 (36.8) for injury-related deaths in 2005. Nonadmitted ED-treated injuries accounted for 67% of YLDs. Estimates for UK DALYs amounted to 1,771,486 (82% due to YLDs), compared with 669,822 (52% due to YLDs) using the GBD approach. Extrapolating patient-derived disability weights to GBD estimates would increase injury-related DALYs 2.6-fold.

**Conclusions:**

The use of disability weights derived from patient experiences combined with additional morbidity data on ED-treated patients and inpatients suggests that the absolute burden of injury is higher than previously estimated. These findings have substantial implications for improving measurement of the national and global burden of injury.

*Please see later in the article for the Editors' Summary*

## Abstract 2

Ronan Lyons and colleagues compared the population burden of injuries using different approaches from the UK Burden of Injury and Global Burden of Disease studies and find that the absolute UK burden of injury is higher than previously estimated.

## Editors' Summary

**Background:**

Injuries—resulting from traffic collisions, drowning, poisoning, falls or burns, and violence from assault, self-inflicted violence, or acts of war—kill more than 5 million people worldwide every year and cause harm to millions more. Injuries account for at least 9% of global mortality and are a threat to health in every country of the world. Furthermore, for every death-related injury, dozens of injured people are admitted to hospitals, hundreds visit emergency rooms, and thousands go to see their doctors by appointment. A large proportion of people surviving their injuries will be left with temporary or permanent disabilities.

The Global Burden of Diseases, Injuries and Risk Factors (GBD) Studies are instrumental in quantifying the burden of injuries placed on society and are essential for the public health response, priority setting, and policy development. Central to the GBD methodology is the concept of Disability Adjusted Life years (DALYs), and a combination of premature mortality, referred to as years of life lost and years lived with disability. However, rather than evidence and measurements, the GBD Study used panel studies and expert opinion to estimate weights and durations of disability. Therefore, although the GBD has been a major development, it may have underestimated the population burden.

**Why Was This Study Done?:**

Accurate measurement of the burden of injuries is essential to ensure adequate policy responses to prevention and treatment. In this study, the researchers aimed to overcome the limitations of previous studies and for the first time, measured the population burden of injuries in the UK using a combination of disability and morbidity metrics, including years of life lost, and years lived with disabilities.

**What Did the Researchers Do and Find?:**

The researchers recruited patients aged over 5 years with a wide range of injuries (including fractures and dislocations, lacerations, bruises and abrasions, sprains, burns and scalds, and head, eye, thorax, and abdominal injuries) from hospitals in four English cities—Swansea, Nottingham, Bristol, and Guildford—between September 2005 and April 2007. The researchers collected data on injury-related mortality, hospital admissions, and attendances to emergency rooms. They also invited patients (or their proxy, if participants were young children) to complete a self-administered questionnaire at recruitment and at 1, 4, and 12 months postinjury to allow data collection on injury characteristics, use of health and social services, time off work, and recovery from injury, in addition to sociodemographic and economic and occupational characteristics. The researchers also used standardized tools to measure health-related quality of life and work problems. Then, the researchers used these patient-reported changes to calculate DALYs for the UK and then extrapolated these results to calculate global estimates.

In the four study sites, a total of 1,517 injured people (median age of 37.4 years and 53.9% male) participated in the study. The researchers found that the vast majority of injuries were unintentional and that the home was the most frequent location of injury. Using the data and information collected from the questionnaires, the researchers extrapolated their results and found that in 2005, there were an estimated 750,999 injury-related hospital admissions, 7,982,947 emergency room attendances, and 22,185 injury-related deaths, translating to a rate per 100,000 of 1,240, 13,339, and 36.8, respectively. The researchers estimated UK DALYs related to injury to be 1,771,486 compared with 669,822 using the GBD approach. Furthermore, the researchers found that extrapolating patient-derived disability weights to GBD estimates would increase injury-related DALYs 2.6-fold.

**What Do These Findings Mean?:**

The findings of this study suggest that, when using data and information derived from patient experiences, combined with additional morbidity data on patients treated in emergency rooms and those, admitted to hospital, the absolute burden of injury is higher than previously estimated. While this study was carried out in the UK the principal findings are relevant to other countries. However, measurement of the population burden of injuries requires access to high quality data, which may be difficult in less affluent countries, and these data rely on access to health facilities, which is often restricted in resource-limited settings. Despite these concerns, these findings have substantial implications for improving measurements of the national and global burden of injury.

**Additional Information:**

Please access these websites via the online version of this summary at http://dx.doi.org/10.1371/journal.pmed.1001140.

- The World Health Organization website provides detailed information about injuries and also details the work of the Global Burden of Disease Study
- The Global Burden of Injury's website is a portal to websites run by groups conducting ongoing research into the measurement of global injury metrics

## Introduction

Quantifying the burden placed on society is an essential component of the public health response to conditions, supporting development of policy, priority setting, and monitoring of interventions [1],[2]. The 1990 Global Burden of Diseases, Injuries and Risk Factors (GBD) Study led the way in developing population burden of disease studies [3]. This seminal study and subsequent publications revealed that injuries accounted for more than 15% of the global disability adjusted life years (DALYs) lost in 1990 and are forecasted to increase to 20% by 2020 [4]–[6]. The methodology has been used widely for the production of estimates of injury-related population burden around the world and is currently being revised with 2005-based estimates due out in 2011 [7]–[14].

Central to the GBD methodology is the concept of DALYs, a combination of premature mortality, termed years of life lost (YLLs), and years lived with disability (YLDs) [15]. YLLs are generated using mortality data. The YLD component requires establishment of disability weights and durations, for different injury types. Disability weights are multiplied by incidence and duration data to generate YLDs. The GBD Study used panel studies and expert opinion to estimate, rather than measure, weights and durations of disability for 33 injury groups [3]. Whilst the GBD was a major development, limited incidence data and the absence of disability weights for many injury types underestimated the population burden of injuries [16]. Accurate measurement of the burden of injuries is essential to ensure adequate policy responses to prevention and treatment.

### Objectives

The UK Burden of Injuries (UKBOI) Study was designed to overcome the limitations of previous studies and to measure the population burden of injuries in the UK for the first time and to compare the population burden of injuries using disability and morbidity metrics from the UKBOI and GBD studies [3],[17].

## Methods

### Study Design

A prospective cohort study, with extrapolation to population-based data was used to establish the burden of injury. The protocol for the design of the UKBOI study has been published previously [17]. Briefly, the main component was a longitudinal study of injured individuals with extrapolation of the impact of their injuries to the UK population using routinely collected data and official statistics on emergency department (ED) attendances, hospital inpatient data, and mortality data, for the year 2005. Injuries were defined using International Classification of Diseases 10 (ICD-10) codes: S00-T73, T75, and T78 [18]. Excluded codes were T79 (certain early complications of trauma), T80–88 (complications of medical and surgical care), and T90–98 (sequelae of injuries and poisoning). For mortality data, the M80–M81 (deaths from osteoporosis) and F10–F19 (deaths from mental and behavioural disorders due to substance abuse) codes were included to ensure that all injury-related deaths due to osteoporosis and poisoning were included [19].

### Setting

The prospective study recruited participants from EDs and hospital inpatients in four UK cities and towns; Swansea, Nottingham, Bristol, and Guildford [17]. Multiple sources of national and regional routine data were used to extrapolate results to the UK population.

### Participants

Inclusion criteria were: participants were patients aged 5 y and over attending EDs or admitted to hospital in the four UK centres with a wide range of injuries, including fractures/dislocations, lacerations, bruises/abrasions, sprains, burns/scalds, and head, eye, thorax, and abdominal injuries (see Table 1 and published protocol [17]). The injuries had to have occurred within 2 wk for ED-treated patients and 4 wk for hospital-admitted patients. Patients had to be able to give consent and complete questionnaires or to have a suitable proxy who could assent to their inclusion and agree to complete future questionnaires.

**Table 1:** Number (%) of cases in UKBOI study by injury group using the 13-group classification.

Type of Injury	*n* Most Severe Injury (%)	*n* All Injuries (%)
Skull-brain injury	19 (1.3)	32 (1.8)
Facial fracture, eye injury	51 (3.4)	62 (3.4)
Spine, vertebrae injury	39 (2.6)	45 (2.5)
Internal organ injury	9 (0.6)	26 (1.4)
Upper extremity fracture	320 (21.1)	355 (19.6)
Upper extremity, other injury	104 (6.9)	119 (6.6)
Hip fracture	65 (4.3)	66 (3.6)
Lower extremity, other fractures	320 (21.1)	352 (19.4)
Lower extremity, other injuries	158 (10.4)	169 (9.3)
Superficial injury, open wounds	312 (20.6)	429 (23.7)
Burns	53 (3.5)	62 (3.4)
Poisonings	1 (0.0)	1 (0.0)
Other injury	66 (4.4)	93 (5.1)
**Total**	**1,517**	**1,811**

Source: [21].

Exclusion criteria were: children under 5 y (due to lack of suitable measurement instruments), those without permanent UK addresses, those who could not give consent and were without a suitable proxy, and those suffering from stings or insertion of foreign bodies in the ear.

Incentives were used to maximise recruitment and retention (£2 vouchers and a 1∶100 chance of a £100 raffle prize at each follow-up point).

Participants were recruited between September 2005 and April 2007. They were followed up until recovery or 12 mo, whichever occurred soonest. Additionally, censoring of participants occurred at refusal to complete the follow-up or death. Two reminders were sent to participants, with participants able to respond even if a previous time point was missed. Participants who did not respond to the full questionnaire were sent a shortened questionnaire, which asked whether they had recovered or not from their injuries. This study received multi-centre research ethics approval from Dyfed Powys National Health Service Local Ethics committee (number 05/WMW01/23). Sequential patients meeting the inclusion criteria and providing informed consent were recruited.

### Variables

Data were collected by self-administered questionnaires at recruitment, and at three fixed times, 1, 4, and 12 mo postinjury, comprising data on socio-demographic, economic, and occupational characteristics, data on injury characteristics, use of health and social services, time off work, recovery from injury, and standardised tools to measure health-related quality of life (HRQL) EQ-5D [20], Health Utilities Index (HUI) [21], PedsQL [22],and work problems (Work Limitations Questionnaire) [23]. Data were also collected on injury mortality, hospital admissions, and ED attendances as described below.

### Bias

Sequential patients meeting the inclusion criteria and providing written informed consent were recruited. In order to prevent biases due to inability to consent to participation (e.g., more serious injuries or those in substantial pain) the inclusion criteria allowed for recruitment up to 2 wk postinjury for ED-treated injuries only and 4 wk for those individuals who were hospitalised. Participants who were unable to provide consent but had a suitable proxy who could assent on their behalf were included in the overall study but were excluded from this component as quality of life measures cannot reliably be obtained by proxy [24]–[27].

The impact of participation bias was assessed by comparing subsequent health service use between recruited participants and potential participants (eligible but not recruited and with similar injuries) in the three most common categories of injury (upper extremity fractures, lower extremity fractures, and superficial injuries and open wounds), subdivided by admitted and nonadmitted patient status. Sensitivity analyses were undertaken to evaluate the effect of losses to follow-up on population estimates of the burden and to assess the impact of response biases on EQ-5D summary scores at different time points. Analysis of variables associated with response at 1 mo was undertaken but not at 4 and 12 mo as it would not be possible to determine whether variables were associated with response or earlier recovery at these time points. Analysis of the impact of missing values on EQ-5D summary scores at different time points was undertaken for a range of assumptions: (a) analysis of complete cases only; (b) analysis using baseline EQ-5D for missing cases if they had returned to normal; (c) analysis using last EQ-5D value when reported recovery for missing cases if they had returned to normal; and (d) analysis using multiple imputation of EQ-5D values in all nonresponders, including recovered cases.

### Study Size

The planned study size of 1,333 (334 per centre) was designed to recruit a minimum of approximately 15–20 per age-specific injury category (see protocol [17]). This number was based on pragmatic grounds, reflecting injury incidence, a feasible recruitment time period, and available research funding rather than a formal statistical sample size calculation.

### Data Sources/Measurement

#### Mortality data

Population mortality data are required to calculate the YLL component of DALYs. Tabulated data on registered deaths in 2005 were available separately for England and Wales, Scotland, and Northern Ireland, and individual-level data were available for Wales. These data were combined to produce population-level mortality data for the UK [17].

#### Hospital admission and ED data

The incidence of nonfatal injuries is necessary to quantify the YLD component of DALYs. Population data on incidence of nonfatal injuries are not available in the UK, as elsewhere, and individual-level data on attendance for treatment at free to use National Health Service EDs and admission to hospital were used with population demographic data to estimate injury incidence. Individual-level data for ED attendances were obtained from five of 13 EDs in Wales and extrapolated to provide UK estimates using a previously developed extrapolation factor [28]. Extrapolating from a sample of EDs to provide a national estimate of injury incidence is a challenging task due to difficulties in assigning catchment areas to derive appropriate denominator data [29]. Five of 13 Welsh EDs participated in an injury surveillance system in 2004. Information on aggregate counts of total attendances at all EDs is available from a Welsh Government system [28]. Comparison between the detailed information from the five hospitals (126,557 injury-related attendances among 232,211 total attendances in 2004) and the aggregate information on all attendances for all hospitals (755,070) produced an extrapolation factor of 3.25 to multiply the cases from the five hospitals to derive incidence numbers and rates for Wales and the UK, using national population denominator data [28]. One approach to validating the extrapolation factor is to compare observed and expected numbers of cases in inpatient data where the expected number comes from the sample of ED cases and the observed number from nationally collected inpatient data. However, many factors affect the likelihood of hospital admission following an injury, the majority unrelated to the severity of the injury [30]. Since ED systems do not routinely collect injury severity measures it is difficult to determine what categories of serious injuries always lead to admission, with the exception of hip fractures [31]. The 3.25 extrapolation factor estimated that there would be 4,071 hip fractures from the ED data for Wales, which compares with an observed of 4,058 recorded in the inpatient database, providing confidence in the accuracy of the extrapolation [28].

Individual-level admissions data for Wales and England were obtained from the Patient Episode Database for Wales (PEDW) and the Hospital Episode Statistics (HES) databases. Inpatient data for Scotland and Northern Ireland (11.3% of the UK population) were not available within the resources of the study and the UK incidence of hospital admissions was extrapolated from the England and Wales rates.

#### Development of the injury weights for injury groups for YLD calculation

The ED and ICD-10 codes were mapped to the 13, 33, and 44 nature of injury categories used in the GBD Study and in previous Dutch burden of injury studies [3],[32],[33]. A greater proportion of ED cases (91%) and inpatient cases (87%) could be mapped to the Dutch 13-group classification [32], compared with 67% and 78%, respectively, for the 33 categories of the GBD study [3], and 66% and 58% for the 44 categories in the Dutch panel study [33].

The primary outcome measure to derive disability weights was the EQ-5D HRQL measure [20], recommended for injury disability studies [34]. The EQ-5D contains five questions, producing up to 243 health state permutations. These permutations are combined with population preference values from expert-panel–based health state valuation and modelling to yield a utility score ranging from <0 (representing a state worse than death) through to 1 (1 implies perfect health), with a score of zero representing a state equivalent to death [35]. The UK EQ-5D tariffs were used to calculate the utility score [36]. The EQ-5D was administered at recruitment (baseline), and at 1, 4, and 12 mo postinjury as described above. A retrospective assessment of preinjury status was obtained at baseline by asking the participant to complete the EQ-5D considering the day prior to injury.

In the context of this study the disability weights represent the difference in EQ-5D utility summary scores derived from subtracting post from preinjury scores in participants who reported at follow-up that they were still affected by their injury. Preinjury EQ-5D scores were also compared to age- and gender-matched population norms for the UK [36]. Once participants reported recovery, no further follow-up was undertaken and recovery was assumed to be complete and permanent. Such participants were given a disability weight of zero at all subsequent follow-up points. When a participant reported recovery between assessment periods, we assumed recovery occurred midway between assessment periods. Time-weighted annualised mean disability weights were calculated for the first year following injury derived from EQ-5D differences from baseline, accounting for the different follow-up periods. The annualised means were derived by estimating the difference in EQ-5D for all participants in any injury group over each of the three time periods (0–1 mo, 1–4 mo, and 5–12 mo), then dividing that by 12 and multiplying by the number of months since the previous follow-up, and summing across the three results to provide a time-weighted average over the first year postinjury. In a small number of cases (*n* = 12 at baseline and *n* = 13 at 12 mo) EQ-5D scores were <0. These scores were included in the analysis. In a number of cases (*n* = 53 at 1 mo) the differences were negative, i.e., higher at follow-up than baseline. These scores were also included in the calculation of average differences for the injury groups. Residual disability at 12 mo, measured by difference between preinjury and 12 mo EQ-5D scores, was considered permanent.

### Statistical Methods

The GBD Study approach for calculating YLLs was adopted, incorporating a 3% discount rate and age weighting, using the published formulae [3]. The published tables for calculating YLLs produced by the GBD were by single year of age; however, UK mortality data were published by 5-y age groups requiring calculations of YLLs based on midpoints within categories. Mean annualised disability weights for the 13 injury groups were calculated by time weighting the responses at the 1-, 4-, and 12-mo data collection points. These weights were then applied to population metrics of injury incidence to calculate YLDs. Population-level YLDs and DALYs were compared using the UKBOI-derived disability weights and the standard GBD approach [3].

In the largest centre (Swansea), data from ED and inpatient injury surveillance systems were available and were used to assess potential response biases by comparing health service use subsequent to the injury. Poisson regression was used to estimate incidence rate ratios (and 95% CI) comparing admission rates, outpatient department attendance rates, and ED attendance rates in the year following injury between participants and nonparticipants. Analysis of variance was used to compare the differences between recorded “preinjury” EQ-5D scores and UK population normative data for the same age group and gender category by duration of delay between recruitment and injury occurrence (0, 1, 2–6, 7–14, and 15–28 d) [36]. Sensitivity analyses were also undertaken, using an extreme case scenario that assumed all nonresponders had recovered at time of last follow-up and suffered no residual disability. Logistic regression was used to identify variables independently associated with response at 1 mo. Repeated measures models (generalised estimating equations) were used to compare response (yes or no) at 1, 4, and 12 mo with those who had recovered at earlier time points counted as responders at later follow-up.

### Deviations from, and Extensions to, the Published Protocol

Target recruitment was exceeded by 184 participants as this was achievable in the recruitment period and increased precision of estimates. The majority of the 1,517 participants (*n* = 1,305, 86%) suffered a single injury. The only notable variations from this figure were for two out of the 13 injury categories: skull/brain injuries where the numbers of single injuries were nine out of 19 (47%); and three out of 9 (33%) for internal organ injuries. Among the ten patients with skull/brain injuries who had more than one injury there were 21 additional injuries, of which 16 were also injuries to the skull/brain. Among the six patients with a primary internal organ injury there were five with fractured ribs, and one case each of tibial, nasal, and orbital floor fractures. Patients were categorised by the most severe injury for analysis purposes. The ICD-10 codes were mapped to the Abbreviated Injury Scale, which provides a severity score for each injury on a scale from 1 (minor) to 6 (maximum) [37].

As the highest proportion of participants' injuries could be matched to the Dutch 13-group classification this categorisation was used to combine routine morbidity data with that from the prospective part of the study to estimate population metrics [32]. Categorisation of injuries for this purpose had not been described in detail in the original published protocol [17].

## Results

### Participants

A total of 1,517 participants participated in the prospective study, with a response rate of 66%. 537 (35.4%) were recruited from the Swansea site, 24.7% from Nottingham, 22.0% from Guildford, and 17.9% from the Bristol site. The median age was 37.4 y (interquartile range 20.6–60.6) and 53.9% were male. The vast majority of injuries were unintentional (91.9%) with 3.5% intentional, 1.9% of uncertain intent, and 2.7% unknown. Home was the most frequent location of injury (34.8%) followed by road traffic injuries (20.6%); 44% were admitted to hospital. Table 1 shows the distribution of cases by the 13 injury categories. It was possible to map all but 66 (4.3%) of the most severe injuries to the 12 specific categories of injury in the 13-category Dutch classification [32]. In contrast it was not possible to map injury data on 267 participants (17.6%) to the 33 GBD categories and 413 (27.2%) to the alternative 44 Dutch disability categories [3],[33].

At 1 mo, 985 participants returned questionnaires (963 full questionnaires and 22 shortened questionnaires) of whom 685 (70%) were still affected by their injury. At 4 mo the figures were 544 (79%) with 375 (69%) still affected, and at 12 mo 323 (86%) with 230 (71%) still affected. 70% (*n* = 691) of “preinjury” assessments of EQ-5D scores were collected within 7 d of the injury. The study sample mean was 3.3% (95% CI 1.9%–4.7%) higher than the UK population, comparing preinjury EQ-5D scores with age- and sex-matched UK population norms [36]. This difference was not affected by variations in time to collect the “preinjury” data (*p* = 0.4). Among the 963 responders with full questionnaires at 1 mo, 863 (90%) answered all EQ5D questions.

### Outcome Data

Table 2 shows the time-weighted disability weights over the 12 mo for hospitalised and nonhospitalised cases. Hospitalised cases had substantially higher weights than those not hospitalised. A common mean weight was applied to a number of the injury groups in which the weights were all low, similar, and involved small case numbers.

**Table 2:** Time-weighted annualised disability weights for the 13-injury group classification by hospitalisation status.

Type of Injury	Hospitalised	Not Hospitalised
Skull, brain injury	0.10	0.007^a^
Facial fracture, eye injury	0.01	0.007^a^
Spine, vertebrae injury	0.34	0.08
Internal organ injury	0.10	—
Upper extremity fracture	0.12	0.07
Upper extremity, other injury	0.16	0.04
Hip fracture	0.24	—
Lower extremity fracture	0.24	0.11
Lower extremity, other injury	0.08	0.05
Superficial injury, open wounds	0.07	0.007^a^
Burns	0.04	0.007^a^
Poisoning	—	—
Other injuries	0.14	0.007^a^

Source: [21].

a A common average disability weight was applied to these groups as the weights were all very low and similar and in some cases the numbers very small. No disability weight was calculated for the one case of poisoning.

A disability weight was not calculated for the single case of poisoning.

### Main Results

#### Injury incidence

Inpatient data for England and Wales identified 665,986 injury-related admissions in the year 2005/2006. Table 3 shows the extrapolated number of admissions (750,999) and ED attendances in the UK by the 13 injury categories. Estimated numbers of hospitalised injuries were subtracted from estimated numbers of ED attendances by each injury group to approximate the number of ED injuries not admitted to hospital. The most frequent causes of admissions were fractures (32.8%), poisoning (18.4%), and superficial injuries/open wounds (19.2%).

**Table 3:** Estimated UK number and population rate of injury admissions, and ED-treated only cases extrapolated from five hospitals in the Welsh Injury Surveillance System in 2005/2006, by the 13 injury group classification.

Injury Group	*n* UK Hospital Admissions	Rate per 100,000	*n* Attendances (WISS)	Extrapolated UK ED Attendances	Rate per 100,000
Skull, brain injury	15,405	25.4	4,907	310,143	518.2
Facial fracture, eye injury	18,062	29.8	867	54,798	91.6
Spine, vertebrae injury	11,706	19.3	7,364	465,436	777.7
Internal organ injury	10,136	16.7	0	0	0.0
Upper extremity fracture	109,859	181.3	13,119	829,176	1,385.5
Upper extremity, other injury	32,852	54.2	11,286	713,323	1,191.9
Hip fracture	65,858	108.7	1,252	79,132	132.2
Lower extremity fracture	70,400	116.2	6,220	393,130	656.9
Lower extremity, other injury	19,454	32.1	15,959	1,008,676	1,685.5
Superficial injury, open wounds	144,390	238.3	48,500	3,065,405	5,122.2
Burns	9,230	15.2	2,252	142,336	237.8
Poisoning	138,559	228.7	3,299	208,511	348.4
Other injury	105,088	173.4	11,279	712,880	1,191.2
**Total**	**750,999**	**1,239.5**	**126,304**	**7,982,947**	**13,339.2**

Source: [21].

WISS, Welsh Injury Surveillance System.

#### YLDs

Table 4 shows the annual UK estimated population-level YLDs (1,450,765) by injury group and hospitalised status using the UKBOI disability weights; 67% were attributed to nonadmitted cases.

**Table 4:** Annual UK-estimated population-level total YLDs using UKBOI disability weights for the 13-injury classification and overall, and proportion from hospitalised cases.

Injury Group	Hospitalised	Nonhospitalised	Total	Percent YLDs Due to Hospitalised Injuries
Skull, brain injury	14,262.90	2,179.24	16,442.14	86.7
Facial fracture, eye injury	257.89	349.61	607.50	42.5
Spine, vertebrae injury	36,813.03	252,251.94	289,064.97	12.7
Internal organ injury	8,001.80	—	8,001.80	100.0
Upper extremity fracture	65,638.98	222,125.93	287,764.91	22.8
Upper extremity, other injury	11,898.50	103,523.00	115,421.50	10.3
Hip fracture	35,173.70	—	35,173.70	100.0
Lower extremity fracture	145,754.52	185,453.20	331,207.72	44.0
Lower extremity, other injury	10,227.94	177,230.48	187,458.42	5.5
Superficial injury, open wounds	41,768.54	24,474.41	66,242.96	63.1
Burns	347.49	1,113.61	1,461.10	23.8
Poisoning	—	—	—	—
Other injury	106,998.67	4,919.31	111,917.98	95.6
**All injuries**	**477,143.97**	**973,620.72**	**1,450,764.69**	**32.9**

Source: [21].

Table 5 shows population-level YLDs for England and Wales by the 33 GBD injury groups using the GBD disability weights for hospital admissions only, as mapping was not possible for nonadmitted patients owing to a lack of ICD10 codes in ED datasets. The number of YLDs was 101,788 for England and Wales and extrapolated to 114,817 for UK hospitalised injuries. This number compared with a figure of 477,144 using the UKBOI disability weights. Assuming that a similar proportion of GBD and UKBOI category cases were admitted, and the relative difference in disability metrics between admitted and nonadmitted cases was the same, there should be an additional 234,284 nonadmitted YLDs in the GBD group, yielding a total of 349,101 for combined admitted and nonadmitted cases. This is a 76% lower estimate than the total of 1,450,765 YLDs using the UKBOI disability weights.

**Table 5:** Annual estimated population-level YLDs following hospital admission for injury in England and Wales by the 33 GBD injury groups, derived from GBD disability metrics.

Injury Group	*n* Hospitalisations in England and Wales^a^	Overall YLDs
GBD1: Fractured skull	2,564	3,812.7
GBD2: Fractured face	12,516	433.4
GBD3: Fractured vertebral column	8,281	257.9
GBD4: Injured spinal cord	2,082	34,707.3
GBD5: Fractured rib or sternum	6,053	119.1
GBD6: Fractured pelvis	9,562	200.0
GBD7: Fractured clavicle, scapula or humerus	23,671	313.0
GBD8: Fractured radius or ulna	53,486	1,070.5
GBD9: Fractured hand bones	20,221	174.6
GBD10: Fractured femur	66,715	7,116.5
GBD11: Fractured patella, tibia or fibula	18,793	484.6
GBD12: Fractured ankle	28,044	575.8
GBD13: Fractured bones in foot	7,234	46.8
GBD14: Other dislocation	2,745	0.0
GBD15: Dislocated shoulder, elbow or hip	6,024	15.5
GBD16: Sprains	2,765	6.8
GBD17: Intracranial injury	11,012	4,226.9
GBD18: Internal injuries	8,985	88.7
GBD19: Open wound	81,912	222.3
GBD20: Injury to eyes	3,491	23,365.1
GBD21: Amputated thumb	403	1,540.3
GBD22: Amputated finger	2,897	7,314.2
GBD23: Amputated arm	18	112.6
GBD24: Amputated toe	77	180.2
GBD25: Amputated foot	7	50.6
GBD26: Amputated leg	39	249.4
GBD27: Crushing	1,875	41.2
GBD28: Burns <20%	23	0.8
GBD29: Burns >20% and <60%	3	17.1
GBD30: Burns >60%	5	22.1
GBD31: Injured nerves	8,149	14,260.8
GBD32: Poisoning	122,807	761.6
GBD33: Other	153,295	0.0
**Total**	**665,754**	**101,788.3**

Source: [3].

a 239 England and Wales hospitalisations were excluded from this analysis because of missing age values.

#### DALYs

In 2005, there were 22,185 injury-related deaths across the UK, producing 320,721 YLLs. The UKBOI adopted the GBD methodology for YLL calculation and so the numbers are the same for both approaches. Summing YLLs and YLDs to produce DALYs revealed that hospital-treated injuries occurring in the UK resulted in an estimated 1,771,486 DALYs using the UKBOI approach. The GBD methodology for YLDs and YLLs for the UK produced 669,822 DALYs, a 62% lower estimate than the UKBOI approach. The 2004 World Health Organization update of the GBD estimated that injury accounted for 12.3% of 1,523 million DALYs [6]. Applying the 2.6-fold relative increase in DALYs from the UKBOI study would increase the global share of injury DALYs to approximately 27%. Sensitivity analyses adopting the conservative approach of assuming all nonresponders had recovered at time of last follow-up and suffered no residual disability reduced the estimate of UK YLDs by 48% (764,845) and DALYs by 39%.

### Representativeness of the Study Sample

The results of the analyses of subsequent health service utilisation between participants and potential participants for the three commonest injury groups are shown in Tables A1–A6 in Text S1. There were no significant differences in rates of subsequent health service use between participants and nonparticipants admitted with upper or lower limb fractures. There were a number of significant differences between participants and nonparticipants with nonadmitted fractures and superficial injuries and wounds, with higher rates of subsequent health service use amongst participants than nonparticipants. For example, for nonadmitted upper arm fractures the incidence rate ratio (IRR) for subsequent admissions was 2.86 (95% CI 1.46–5.52) and 1.40 (95% CI 1.19–1.66) for outpatient attendances. For nonadmitted lower limb fractures the IRR for outpatient attendances was 1.47 (95% CI 1.13–1.90). For nonadmitted superficial injuries and open wounds the IRRs were 2.26 (95% CI 0.98–5.19) for admissions, 2.38 (95% CI 1.13–5.0) for outpatient attendances.

The results of analysis of factors associated with retention at 1 mo are shown in Table A7 in Text S1. Retention was higher for those aged 45–64 y (odds ratio [OR] 2.1) and lower (OR 0.5) for those aged 15–24 y, and lower in the more deprived communities (OR 0.48 in most deprived quintile). ORs for retention of most injury types were higher than for superficial injuries.

The results of different approaches to the analysis of the impact of missing data on EQ-5D summary scores are shown in Table A8 in Text S1. Imputed values were similar to those obtained using substitution methods, including that used for the analyses presented in this paper, but were higher than those based on a complete case analysis with EQ-5D mean summary scores being 3.2% higher at 1 mo, 8.2% higher at 4 mo, and 11.6% higher at 12 mo. The mean annualised disability weight in our main analysis, which assumed that those who had recovered returned to their baseline EQ5D, was −0.10. Imputing values for all nonresponders including recovered cases who were not sent further follow-up questionnaires resulted in a mean annualised disability weight of −0.11.

## Discussion

### Key Results

The results of the UKBOI study show that combining empirical data from injured individuals with multiple sources of incidence data produces much higher estimates of population burden of injuries than the GBD study methodology, the standard to date. A number of reasons are behind this finding, but key amongst them is the issue of substantially higher disability weights derived from reports of injured patients compared with those derived from uninjured panels. Despite a relatively low injury mortality rate based on international comparisons, injuries in the UK result in a substantial population burden in terms of disability and premature mortality [38]. This study is the first, to our knowledge, to report that the burden of injury from disability is larger than that from death.

Valid estimation of the burden of injury is critical for accurate ranking of injury as a global public health issue, prioritisation of prevention efforts, policy development, and health service planning [1],[2]. A notable limitation of the GBD Study methodology was the reliance on panel and expert opinion to derive disability weights rather than using empirical data from injured populations [3],[16]. While other studies have developed alternative disability weights [33],[35] and utilised disability weights derived from patient experiences to estimate YLDs, the UKBOI study has extended the application of patient-derived disability weights and multiple routine data sources to produce what appears to be the most comprehensive population-based burden of injury study to date using the DALY method [17]. We compared results with other published studies producing population-level DALYs for all injuries [6],[8],[10],[11],[13],[14] and used a recent systematic analysis of studies measuring HRQL in general injury populations to ensure we did not miss studies [39].

Combining data from the UKBOI prospective study of injured individuals with the best available routine data on ED and inpatient treatment for injuries and mortality data produced an estimated 1.77 million injury-related DALYs for the UK from injuries occurring in 2005, a rate of 2,924/100,000 population. The DALY total comprised 320,721 YLLs (18%) and 1,450,764 YLDs (82%). Nonhospitalised cases amounted to 973,620 YLDs, 67% of the YLD total. These estimates are substantially higher than published studies using the original GBD methodology [7]–[14]. Table 6 shows the YLL, YLD, and total DALYs per 100,000 population reported by published studies where sufficient information was provided to calculate a rate per 100,000 population. There are difficulties in comparing burden of injury studies as many did not declare whether age weighting and discounting were used. Nevertheless, the YLD estimate from the current study is 2.9 times higher than the next largest estimate from Austria in 1999 and the UKBOI is the first to report that the majority of the injury burden relates to disability rather than death (Table 6). There are several major differences that could explain these findings.

**Table 6:** Estimates of injury-related YLDs, YLLs, YLL;YLD ratio, and DALYs per 100,000 population from published studies and the UKBOI Study.

Country, Year (Reference Number)	YLDs	YLLs	YLL∶YLD Ratio	DALYs
World, 2004 [6]	—	—	—	2,702
Australia, 1996 [8]	263	697	2.7	959
Austria, 1999 [14]	820	1,710	2.1	2,530
Denmark, 1999 [14]	340	1,550	4.6	1,890
Ireland, 1999 [14]	430	1,530	3.6	1,960
Netherlands, 1999 [14]	310	940	3.0	1,260
Norway, 1999 [14]	320	1,410	4.4	1,720
England, 1999 [14]	240	980	4.1	1,220
Wales, 1999 [14]	250	980	3.9	1,230
United States, 1996 [10]	—	—	—	1,450
Iran, 2003 [13]	—	—	—	6,040
South Africa, 2000 [11]	—	—	—	5,348
United Kingdom, 2005, GBD weights	577	526	0.9	1,103
United Kingdom, 2005, UKBOI weights	2,398	526	0.2	2,924

Some publications did not provide results or sufficient data to calculate YLDs or YLLs. The European study of six countries did not provide a separate analysis of mortality for England and Wales [14]. It is unclear whether all studies adjusted for the age weighting and discounting as used in the GBDs study [5].

#### Injury groupings

The current study mapped injuries to the 13-group classification described by Meerding et al. because a sizeable proportion (22%–40%) of injury attendance and admission data cannot be mapped to the 33 categories of the GBD Study or the 44 categories derived by another Dutch panel study, thus excluding these injuries from burden estimates [3],[32],[33]. Although 9% of ED cases and 13% of inpatient cases could not be mapped to specific categories using the 13-group classification, these could still be included in UKBOI YLD estimates under the “other injury” category, ensuring that important injury diagnoses were not lost from burden estimates.

A further related issue is the ability to map data from the longitudinal study to injury categories to derive average disability weights to be applied to the routine data to estimate the population burden. It was possible to map all but 66 (4.3%) of the most severe injuries to the 12 specific categories of injury using the Dutch 13-group classification [32]. In contrast it not possible to map injury data on 267 participants (17.6%) to the 33 GBD categories [3]. There is no GBD disability weight for “other” and, hence, these injuries do not contribute to the calculation of population-level YLDs. The fact that this study shows the difficulties of mapping to the limited GBD categories is useful in highlighting the gaps in the GBD categories and informing their revision.

#### Disability weight generation

Disability weights in the UKBOI study were derived from the experiences of 1,517 injured individuals, while the GBD and Dutch panel study weights were derived from panel valuation exercises [3],[33]. Panel studies require groups without the injury experience to value the impact of the injury using short vignettes of typical impact and duration provided by medical experts [33],[35]. While the Dutch panel study weights have been shown to result in greater YLDs than the GBD study [33], providing an improvement on the original GBD study weights, a comparison of disability weights derived using EQ-5D scores from 1,392 patients with lay panel-derived disability weights found that, for all but one injury health state, the patient-derived weights indicated greater disability than the panel-derived weights [35]. The authors favoured the panel-derived weights, concluding that the patient-derived weights result in “overestimation” of the disability resulting from more minor injuries, due to the potential for reporting bias and differences between self-reported health status and “actual” health status [35]. However, self-reported health status is the patient's health status as the EQ-5D and other measures of HRQL are designed to reflect the patient's perceptions of their health, making patients the most reliable witness of their health status.

Additionally, apart from fundamental differences in deriving values obtained by those who have and have not experienced injuries, there is the possibility that the standard vignettes do not portray an accurate reflection of the impact and duration of injuries, a limitation previously acknowledge by panel-based studies [35]. Summarising the course of an injury in lay terminology, while adequately addressing the variability in recovery that occurs related to personal and injury severity factors, is difficult [35]. The use of lay panels to derive disability weights is also time consuming and expensive, limiting the number of health states for which disability weights can be generated [33],[35].

Comparison of disability weights with other studies is difficult due to differing injury categories. However, the upper and lower extremity fractures subgroups were comparable in the UKBOI, GBD, and Dutch panel studies. The weights for upper extremity fractures were 0.12 and 0.07 for admitted and nonadmitted fractures in the UKBOI study, compared with 0.02 and 0.04 for the GBD and Dutch panel studies (undifferentiated by admission status) [3],[33]. For lower extremity fractures the respective figures were 0.24 and 0.11, compared to 0.02 and 0.06 (admitted cases) [3],[33]. The UKBOI figures are substantially higher than the GBD and Dutch study metrics. Consistent with the previous Dutch study the most likely explanation for this is the discrepancy between the experience of actual patients and that portrayed in the standardised vignettes of the impact and duration of injuries presented to panel studies [35]. Certainly, the duration of impact in such vignettes (often described as a number of weeks) does not appear to accurately reflect the experience of many patients [35].

#### Long term disability

Related to the derivation of disability weights are differences in the proportion of cases with life-long impact. The GBD Study assumed that only certain injury categories had life-long impact and these were mainly the more severe and rarer categories, such as major burns, amputations, and spinal cord injuries [3]. Head injuries and hip fractures were the exception for more common injuries and it was assumed that 15% of cases of fractured skulls, 5% of intracranial injuries, and 5% of hip fractures had life-long impact. In the UKBOI study, disability at 12 mo was assumed to be life long, which is consistent with existing literature for injury studies [40],[41]. In the UKBOI study, 15% of recruited participants were still affected at 12 mo for all injuries; 32% of hip fractures, and 23% of other lower extremity fractures. The high rate of residual impairment at 12 mo and the associated assumption of life-long disability has a substantial effect on population metrics, with 62% of population YLDs in the UKBOI occurring after the first 12 mo. There are a couple of studies limited to major trauma patients, which show some improvement in outcomes after 1 y, another that shows worsening of health status in the longer term; but it is not possible to use these selected groups to refine our estimates across a broad range of injuries [42]–[44]. There is a need for further longer term studies to clarify longer term outcomes.

#### Data sources and quality

Another difference between the UKBOI study and others was the greater availability of data on injury incidence, a well-recognised problem in burden of disease measurement [45]. The GBD study suffered substantially from a lack of morbidity data and thus could only produce estimates for major regions and economies of the world [3]. The Australian Burden of Disease and Injury Study used the GBD approach and estimated that injuries accounted for 7.1% of the 2.5 million DALYs in Australia in 1996, a population rate of 9.7/1,000, contrasting with the UKBOI estimate of 29.2/1,000 population [8]. Whether the difference is due to the more extensive morbidity data or the use of empirical rather than the GBD Study disability weights used in the Australian Study is unclear. A study of six western European countries adopted the GBD weights and reported a DALY rate of 12.2/1,000 for England and Wales, much lower than the UKBOI estimate [14]. Whilst there were some differences in the handling of morbidity data, the use of the GBD disability weights is likely to be the primary reason for the difference in population-level DALYs observed. The 1996 US Burden of Disease and Injury Study (USBODI) derived DALYs from a mixture of national surveys, hospital discharge data, disease registers, and extrapolation of YLDs from YLD∶YLL ratios for the established market economy (EME) countries from the GBD Study where data were unavailable [4],[10]. This study estimated a US population rate of injury DALYs of 14.5/1,000, again considerably lower than the UKBOI estimates. The difference is likely due to the use of the GBD weights, but insufficient published methodological information precludes a detailed comparison.

Both the UKBOI Study and a US study on injury costs demonstrate the importance of ED data in estimating aspects of the population burden of injuries [9]. Whilst disability weights in hospitalised cases in the UKBOI were two to ten times greater than in nonhospitalised cases (Table 2), the greater incidence of nonhospitalised cases meant that nonhospitalised cases contributed two-thirds of YLDs.

### Limitations

As with every study, case selection and losses to follow-up may have introduced bias.

Recruiting patients to studies in emergency settings is challenging given ethical and other constraints [46]. The ethics committee that approved this study required potential participants to first be approached by health service staff who then sought consent for them to be approached by the researchers. The researchers were based in the ED and hospital wards. The response rate (66%) to being approached could be monitored only for the first 2 wk of the study because of the logistical difficulties of this requirement. Whilst this response rate is higher than in other studies it is far from optimal. Undoubtedly, the response rate would be higher if researchers were able to approach patients directly. Despite the use of £2 vouchers and £100 raffle prizes as incentives at each follow-up point, 20%–35% of respondents were lost at different follow-up points. However, the retention rate at 1 mo (65%) was higher than the use of mail-out recruitment in the previous Dutch study (37% at 2.5 mo), whereas losses at other time points were similar between studies [32]. However, the findings derived from the record linkage follow-up component at the Swansea site suggest that there was no major bias in the representativeness of admitted patients recruited to the study, but that nonadmitted participants tended to have more severe injuries than nonparticipants, as judged by differences in subsequent health service utilisation in comparator groups. Further research is needed to quantify the strength of the relationship between health service utilisation and disability before such information could be used to adjust results quantitatively.

We also attempted to quantify the potential impact of losses to follow-up. Adopting the very conservative approach of assuming all losses to follow-up were fully recovered would reduce our estimate of population-level YLDs by 48% and DALYs by 39%, but our estimates would still be 1.6-fold higher than those estimated using the GBD approach [4].

A variety of approaches were used to model the effects of missing data (Table A8 in Text S1). Imputed values were similar to those obtained using substitution methods but were higher than those based on a complete case analysis with EQ-5D mean summary scores being 3.2% higher at 1 mo, 8.2% higher at 4 mo, and 11.6% higher at 12 mo. There was little difference in the mean annualised disability weights resulting from assuming that those who had recovered had returned to their baseline EQ5D (−0.10) and from multiple imputation of missing values (−0.11). This finding suggests taking account of missing data would not result in substantial under- or overestimation of the burden of injury.

A further methodological choice made was to limit follow-up at additional time points to only those who reported disability at the previous time point. A previous study found deterioration in health status over time for some patient groups, but failed to quantify the proportion of patients who deteriorated and recommended cautious interpretation of these findings because of the inability to determine the impact of comorbidities and other factors on the health status of patients [47]. Where deterioration occurs, it is difficult to ascribe this to the original injury, a subsequent injury, or the development of a related or unrelated comorbidity. Overall, the impact on the disability weights and overall estimates is unlikely to be large, but warrants noting as a limitation of the study.

We also lacked individual level routine data for various aspects of the study. Estimates for ED-treated injuries were based on data from only five Welsh hospitals. Whilst the agreement between observed and expected numbers for hip fractures suggests the wider extrapolation was accurate, the possibility that injury patterns in Wales differ from the rest of the UK remains. However, as mortality rates differ by only about 5%, the bias is unlikely to be major. Whilst the inclusion of limited ED data was a strength of this study, many people with injuries do not seek ED treatment despite being associated with substantial disability [48]. Thus, the population burden will be even greater than our estimates, an issue with any study that uses medically attended injuries to measure incidence. However, response and recall biases inherent in surveys means that it may not be possible to accurately measure the true population incidence of injuries [49].

Poisoning is included in the list of ICD codes used to define injuries for international studies [18],[19]. We excluded disability from poisoning in the calculation of YLDs (but not YLLs) as there was only a single case included in the longitudinal study. Table 5 shows that poisoning accounted for 122,807 hospital admission in England and Wales in 2005, 18.4% of the total for injuries and poisoning. It is difficult to assess the effect of this exclusion but it is probably not too large in the UK context where the most common poisoning agents (e.g., paracetamol) rarely lead to mortality or long term physical damage [50]. This situation would not be the case in other settings where the use of more corrosive and toxic agents is much more prevalent.

As in many studies it was not possible to accurately determine whether death was a reason for nonresponse. In the Swansea site where a record linkage system was subsequently put in place, we recorded seven deaths within 12 mo among 468 traceable participants. Hence we would expect about 22 to 23 deaths in the entire study; however, as these are not detectable amongst all the cases of nonresponse they were treated as losses to follow-up. This finding highlights another important methodological issue common to all burden of injury studies. Measurement of injury-related DALYs assumes that population-level estimates of injury-related mortality are accurate. However, we know that mortality rates are elevated for long periods postinjury and these delayed injury-related deaths are frequently attributed to other conditions [51]. Thus, routine mortality data underestimate injury-related YLLs.

Measuring the population burden of injuries is a complex task, made particularly difficult by the heterogeneity of injury populations with respect to injuries sustained, severity, and demographic profiles. Deriving disability weights for all injury diagnoses is not feasible for panel or empirical data studies. For example, recruiting just 12 cases for each 5-y age and gender group, for each of the 1,278 ICD-10 injury diagnostic codes requires a sample size of 552,096 cases. In order to produce metrics categorisation of injuries is necessary into logical but possibly heterogeneous groups, and there will inevitably be a considerable degree of heterogeneity in the 13-group categorisation used in this study [32]. At the same time, use of greater number of injury groups, as in the GBD and Dutch panel study, replaces one problem with another in excluding high proportions of patients with injuries [3],[33]. In our study, the disability weights were generated on the basis of relatively small numbers of patients in each injury category. Each disability weight is essentially a time-weighted average of individual responses, providing a challenge for calculating the precision of the estimate of the time-weighted annualised disability weights. While measures of precision could be calculated for each time point (i.e., 1 mo, 4 mo, 12 mo), extrapolation to the time-weighted disability weight was not possible. Work on developing disability weights for a larger group of injuries is being undertaken as part of the GBD revision process. Prospective outcome studies in injury populations are still rare and more are required. Given sample size requirements, cost, and logistical challenges, there is also a need for meta-analyses of the existing individual-level data around the world.

Injured patients consistently report preinjury HRQL above population norms, although this is usually collected retrospectively [52],[53]. There is potential for recall bias in the retrospective reporting of preinjury HRQL. However, a clear consensus has not been reached as to whether the difference between population norms and retrospectively recalled scores is the result of response shift caused by the injury, or simply the injury population being healthier than population norms. A previous study has shown that the HRQL scores for injured patients reporting that they had recovered were consistent with their retrospective preinjury scores [54]. In the current study, whilst preinjury HRQL scores were 3% higher than age- and gender-weighted norms the difference was not affected by the timing of assessment of “preinjury” status within a 4-wk period postinjury and time postinjury. This finding suggests little evidence of bias in the reporting of retrospective preinjury HRQL scores when collected within weeks of injury.

Numerous instruments are available for assessing HRQL following injury. The UKBOI study used the EQ-5D, which has been recommended for injury studies [34]. A comparison of the EQ-5D and the Health Utilities Index (HUI) suggests the performance of these instruments differs according to the patient population study with the authors recommending the HUI over the EQ-5D [55]. However, the differences were noted to be small and inconsistent in pattern, and the EQ-5D resulted in higher completion rates and less expense to implement, supporting the use of the EQ-5D [56].

### Interpretation

There are many challenges in estimating the population burden of injuries. The results of this study demonstrate that using disability weight data from a prospective study of injured patients with additional morbidity data sources produces estimates of the population burden of injuries that are considerably higher than previous estimates derived from the standard GBD approach [3],[4]. Whist considerable uncertainties remain, our best estimate is that injury-related DALYs are 2.6 times greater than previously thought, and even if we accept a very conservative approach of assuming no residual disability in all losses to follow-up the population estimate would be 1.6 times earlier estimates.

### Generalizability

Whilst this study was carried out in the UK, the principal findings are relevant across the globe. Measurement of the population burden of injuries requires access to high quality morbidity data, which must include sources other than hospital admission data. This need will be particularly the case in less affluent countries where there is often very restricted access to health care facilities. This point has been previously highlighted by the Global Burden of Diseases Injury Expert Group, which published a call in this journal for better access to morbidity and mortality data to improve estimates of the global burden [44]. It is also clear that disability weights and durations derived from injured patients are at considerable variance from those derived from expert panels and that decisions on which to use will fundamentally affect the magnitude of the burden of injury.

Our results suggest that if the pattern of underestimation seen in the UK was mirrored across the world then injuries may account for up to a quarter of global DALYs rather than a sixth as previously estimated [6]. This estimate is not precise as it is sensitive to the relative contribution of the mortality and morbidity components of DALYs and also to improvements and refinements in data and outcome metrics for other diseases and disorders, which might change the overall attribution of DALYs between injury and illness. However, undoubtedly the global proportion of DALYs from injury is larger than previously estimated. Accurate measurement of the burden of injuries is essential to ensure appropriate policy responses to prevention and treatment. There is already evidence that policy and research responses to injury are grossly inadequate, based upon the previous estimates of the burden [57],[58].

## Supporting Information

Text S1 Additional tables.
(DOC)

## Abbreviations

DALY: disability adjusted life year
ED: emergency department
GBD: global burden of diseases
HRQL: health-related quality of life
ICD-10: International Classification of Diseases 10
UKBOI: UK Burden of Injury
YLD: years lived with disability
YYL: years of life lost
