# Supplementary material for: Measuring the Population Burden of Injuries—Implications for Global and National Estimates: A Multi-centre Prospective UK Longitudinal Study
Source: PLoS Med. 2011 Dec 6;8(12):e1001140. doi: 10.1371/journal.pmed.1001140 (PMC3232198; doi:10.1371/journal.pmed.1001140)
Supplement: Text S1 — Additional tables. (DOC) [file pmed.1001140.s001.doc]

SUPPORTING INFORMATION

Text S1: Additional tables

The supporting text includes additional tables comparing participants and potential participants with three categories of injury by admission status from the Swansea site (A1-A6) and two tables dealing with analysis of missing values (A7-A8).

Tables A1-A6 provide a comparison of demographic factors including deprivation fifths (quintiles), and subsequent health services utilisation in the twelve months following injury between those recruited to the study and all those with similar injuries attending the Swansea site. The three injury categories analysed are upper extremity fractures, lower extremity fractures, and superficial injuries/open wounds used in the Dutch burden of injuries study [32].

Table A1. Characteristics and injury rates of admitted participants and non participants with upper extremity fractures

|  | Participants (n=15) | Non participants (n=819) | Significance |
| --- | --- | --- | --- |
| Age (median (IQR)) | 49 (23, 68) | 31 (12, 61) | Z=-1.54, p=0.12 |
| Male (%) | 8 (53.3) | 454 (55.4) | Χ^2^_(1)_=0.03, p=0.87 |
| Deprivation quintiles*  1 (Least deprived)  2  3  4  5 (Most deprived) | 2 (13.3)  2 (13.3)  2 (13.3)  6 (40.0)  3 (20.0) | 134 (16.8) [19]  139 (17.4)  140 (17.5)  171 (21.4)  216 (27.0) | Χ^2^_(1)_=0.17 p=0.68 |
|  |  |  | Incidence rate ratio (95% CI) adjusted for age, sex and deprivation. |
| Annual in patient admission rate/1000 (Poisson exact 95% CI) | 1400.0 (866.6, 2140.0) | 1225.9 (1151.2, 1304.1) | 1.14 (0.74, 1.76) p=0.56 |
| Annual outpatient attendance rate/1000 (Poisson exact 95% CI) | 5200.0 (4110.4, 6489.8) | 4140.4 (4002.2, 4282.2) | 1.23 (0.95, 1.59) p=0.12 |
| Annual ED attendance rate/1000 (Poisson exact 95% CI) | 333.3 (108.2, 777.9) | 399.3 (357.2, 445.0) | 0.82 (0.28, 2.38) p=0.71 |

* Quintiles of Townsend score for Wales based on 2001 Census.

[ ] missing values

Table A2. Characteristics and injury rates of non admitted participants and non participants with upper extremity fractures

|  | Participants (n=75) | Non participants (n=5569) | Significance |
| --- | --- | --- | --- |
| Age (median (IQR)) | 32 (13, 63) | 23 (12, 48) | Z=-2.37, p=0.02 |
| Male (%) | 36 (48.0) | 3081 (55.3) | Χ^2^_(1)_=1.61, p=0.21 |
| Deprivation quintiles*  1 (Least deprived)  2  3  4  5 (Most deprived) | 19 (25.7) [1]  12 (16.2)  11 (14.9)  10 (13.5)  22 (29.7) | 1078 (19.8) [113]  960 (17.6)  977 (17.9)  926 (17.0)  1515 (27.8) | Χ^2^_(1)_=0.33 p=0.57 |
|  |  |  | Incidence rate ratio (95% CI) adjusted for age, sex and deprivation. |
| Annual in patient admission rate/1000 (Poisson exact 95% CI) | 213.3 (121.9, 346.4) | 72.2 (65.3, 79.6) | 2.86 (1.46, 5.62) p=0.002 |
| Annual outpatient attendance rate/1000 (Poisson exact 95% CI) | 3520.0 (3108.2, 3971.2) | 2450.5 (2409.6, 2492.0) | 1.40 (1.19, 1.66) p<0.001 |
| Annual ED attendance rate/1000 (Poisson exact 95% CI) | 773.3 (587.2, 999.7) | 525.4 (506.5, 544.8) | 1.50 (1.02, 2.20) p=0.04 |

* Quintiles of Townsend score for Wales based on 2001 Census.

[ ] missing values

Table A3. Characteristics and injury rates of admitted participants and non participants with lower extremity fractures

|  | Participants (n=51) | Non participants (n=623) | Significance |
| --- | --- | --- | --- |
| Age (median (IQR)) | 44 (25, 55) | 46 (23, 72) | Z=1.06, p=0.29 |
| Male (%) | 31 (60.8) | 334 (53.6) | Χ^2^_(1)_=0.98, p=0.32 |
| Deprivation quintiles*  1 (Least deprived)  2  3  4  5 (Most deprived) | 12 (23.5)  10 (19.6)  10 (19.6)  9 (17.7)  10 (19.6) | 95 (15.9) [24]  104 (17.4)  117 (19.5)  120 (20.0)  163 (27.2) | Χ^2^_(1)_=2.84 p=0.09 |
|  |  |  | Incidence rate ratio (95% CI) adjusted for age, sex and deprivation. |
| Annual in patient admission rate/1000 (Poisson exact 95% CI) | 1333.3 (1035.4, 1690.3) | 1361.2 (1271.1, 1455.9) | 0.98 (0.76, 1.26) p=0.87 |
| Annual outpatient attendance rate/1000 (Poisson exact 95% CI) | 5647.1 (5013.6, 6338.4) | 4725.5 (4556.3, 4899.4) | 1.09 (0.91, 1.30) p=0.36 |
| Annual ED attendance rate/1000 (Poisson exact 95% CI) | 529.4 (348.9, 770.3) | 319.4 (276.6, 367.0) | 1.49 (0.89, 2.49) p=0.13 |

* Quintiles of Townsend score for Wales based on 2001 Census.

[ ] missing values

Table A4. Characteristics and injury rates of non admitted participants and non participants with lower extremity fractures

|  | Participants (n=46) | Non participants (n=2721) | Significance |
| --- | --- | --- | --- |
| Age (median (IQR)) | 40 (22.3, 58.3) | 29 (15,50) | Z=-2.22, p=0.03 |
| Male (%) | 17 (37.0) | 1387 (51.0) | Χ^2^_(1)_=3.56, p=0.06 |
| Deprivation quintiles*  1 (Least deprived)  2  3  4  5 (Most deprived) | 17 (37.0)  8 (17.4)  5 (10.9)  7 (15.2)  9 (19.6) | 527 (19.8) [54]  492 (18.5)  486 (18.2)  474 (17.8)  688 (25.8) | Χ^2^_(1)_=4.85 p=0.03 |
|  |  |  | Incidence rate ratio (95% CI) adjusted for age, sex and deprivation. |
| Annual in patient admission rate/1000 (Poisson exact 95% CI) | 173.9 (75.1, 342.7) | 64.7 (55.5, 75.0) | 2.49 (0.86, 7.18) p=0.09 |
| Annual outpatient attendance rate/1000 (Poisson exact 95% CI) | 3260.9 (2759.9, 3826.5) | 2184.1 (2128.9, 2240.4) | 1.47 (1.13, 1.90) p=0.004 |
| Annual ED attendance rate/1000 (Poisson exact 95% CI) | 500.0 (317.0, 750.2) | 460.9 (435.7, 487.1) | 1.29 (0.75, 2.22) p=0.35 |

*Quintiles of Townsend score for Wales based on 2001 Census.

[ ] missing values

Table A5. Characteristics and injury rates of admitted participants and non participants with superficial injuries and open wounds

|  | Participants (n=16) | Non participants (n=957) | Significance |
| --- | --- | --- | --- |
| Age (median (IQR)) | 36 (21.8, 46.5) | 31 (19, 50) | Z=-0.71, p=0.48 |
| Male (%) | 13 (81.3) | 672 (70.2) | Χ^2^_(1)_=0.92, p=0.34 |
| Deprivation quintiles*  1 (Least deprived)  2  3  4  5 (Most deprived) | 4 (25.0)  1 (6.3)  4 (25.0)  2 (12.5)  5 (31.3) | 143 (15.3) [25]  161 (17.3)  182 (19.5)  193 (20.7)  253 (27.2) | Χ^2^_(1)_= 0.05, p=0.82 |
|  |  |  | Incidence rate ratio (95% CI) adjusted for age, sex and deprivation |
| Annual in patient admission rate/1000 (Poisson exact 95% CI) | 1375.0 (861.7, 2081.8) | 1148.4 (1081.5, 1218.4) | 1.21 (0.79, 1.85) p=0.38 |
| Annual outpatient attendance rate/1000 (Poisson exact 95% CI) | 4000.0 (3080.5, 5107.9) | 2621.7 (2520.1, 2726.4) | 1.43 (0.93, 2.20) p=0.10 |
| Annual ED attendance rate/1000 (Poisson exact 95% CI) | 937.5 (524.7, 1546.3) | 383.5 (345.3, 424.8) | 3.27 (1.22, 8.72) p=0.02 |

* Quintiles of Townsend score for Wales based on 2001 Census.

[ ] missing values

Table A6. Characteristics and injury rates of non admitted participants and non participants with superficial injuries and open wounds

|  | Participants (n=75) | Non participants (n=16062) | Significance |
| --- | --- | --- | --- |
| Age (median (IQR)) | 35 (23, 58) | 26 (15, 47) | Z=-3.02, p=0.003 |
| Male (%) | 42 (56.0) | 9425 (58.7) | Χ^2^_(1)_=0.22, p=0.64 |
| Deprivation quintiles*  1 (Least deprived)  2  3  4  5 (Most deprived) | 16 (21.3)  19 (25.3)  11 (14.7)  8 (10.7)  21 (28.0) | 2673 (17.0) [311]  2692 (17.1)  2938 (18.7)  3013 (19.1)  4435 (28.2) | Χ^2^_(1)_= 2.35, p=0.13 |
|  |  |  | Incidence rate ratio (95% CI) adjusted for age, sex and deprivation |
| Annual in patient admission rate/1000 (Poisson exact 95% CI) | 146.7 (73.2, 262.4) | 54.5 (50.9, 58.2) | 2.26 (0.98, 5.19) p=0.05 |
| Annual outpatient attendance rate/1000 (Poisson exact 95% CI) | 880.0 (680.6, 1119.6) | 354.4 (345.3, 363.8) | 2.38 (1.13, 5.00) p=0.02 |
| Annual ED attendance rate/1000 (Poisson exact 95% CI) | 720.0 (540.9, 939.4) | 514.9 (503.9, 526.2) | 1.40 (0.95, 2.07) p=0.09 |

* Quintiles of Townsend score for Wales based on 2001 Census.

[ ] missing values

Table A7. Results of logistic regression model of variables independently associated with response at 1 month.

| Variable | Odds ratio | 95% CI | P value |
| --- | --- | --- | --- |
| **Centre** |  |  |  |
| Bristol | 1.00 |  |  |
| Swansea | 0.84 | (0.59 to 1.18) | 0.315 |
| Nottingham | 1.50 | (1.02 to 2.19) | 0.037 |
| Surrey | 0.74 | (0.50 to 1.10) | 0.142 |
| **Age-group** |  |  |  |
| 5-14 | 1.00 |  |  |
| 15-24 | 0.50 | (0.33 to 0.74) | 0.001 |
| 25-44 | 0.77 | (0.52 to 1.13) | 0.186 |
| 45-64 | 2.10 | (1.37 to 3.2) | 0.001 |
| 65+ | 1.93 | (1.25 to 2.97) | 0.003 |
| **Quintile of deprivation** |  |  |  |
| 1 | 1.00 |  |  |
| 2 | 1.07 | (0.74 to 1.56) | 0.716 |
| 3 | 0.66 | (0.46 to 0.95) | 0.026 |
| 4 | 0.44 | (0.30 to 0.64) | <0.001 |
| 5 | 0.48 | (0.32 to 0.72) | <0.001 |
| **Injury category [32]** |  |  |  |
| skull-brain injury | 1.35 | (0.43 to 4.23) | 0.605 |
| facial fracture/eye | 1.19 | (0.60 to 2.34) | 0.623 |
| spine/vertebrae | 4.10 | (1.57 to 10.65) | 0.004 |
| internal organ | 1.24 | (0.29 to 5.38) | 0.771 |
| upper extremity, fracture | 1.93 | (1.34 to 2.78) | <0.001 |
| upper extremity, other | 2.14 | (1.27 to 3.62) | 0.004 |
| hip fracture | 1.33 | (0.68 to 2.60) | 0.403 |
| lower extremity, fracture | 1.38 | (0.96 to 1.98) | 0.079 |
| lower extremity, other | 1.61 | (1.04 to 2.47) | 0.032 |
| superficial injury, open | 1.00 |  |  |
| burns | 2.51 | (1.26 to 4.98) | 0.009 |
| poisonings | - |  |  |
| other injury/none | 1.26 | (0.66 to 2.39) | 0.483 |

Variables that did not have a significant association at P<0.05 were removed from the model.

Table A8. Impact of different approaches for handling missing values on EQ-5D summary scores at different time points. Results are presented for mean EQ-5D summary scores, standard errors of the mean (SE), standard deviation (SD), median, inter quartile range (IQR) and count (sample size).

Approach 1: analysis of complete cases only

|  | Baseline | 1 month | 4 months | 12 months |
| --- | --- | --- | --- | --- |
| Mean | 0.91 | 0.61 | 0.73 | 0.70 |
| SE (mean) | 0.01 | 0.01 | 0.01 | 0.02 |
| SD | 0.21 | 0.33 | 0.29 | 0.29 |
| Median | 1.00 | 0.69 | 0.80 | 0.73 |
| IQR | (0.85 to 1.00) | (0.36 to 0.80) | (0.62 to 1.00) | (0.62 to 1.00) |
| Count | 1481 | 863 | 460 | 266 |

Approach 2: analysis using baseline EQ-5D for missing cases if they had returned to normal

|  | Baseline | 1 month | 4 months | 12 months |
| --- | --- | --- | --- | --- |
| Mean | 0.91 | 0.61 | 0.81 | 0.85 |
| SE (mean) | 0.01 | 0.01 | 0.01 | 0.01 |
| SD | 0.21 | 0.33 | 0.26 | 0.25 |
| Median | 1.00 | 0.69 | 1.00 | 1.00 |
| IQR | (0.85 to 1.00) | (0.36 to 0.80) | (0.71 to 1.00) | (0.74 to 1.00) |
| Count | 1481 | 863 | 749 | 715 |

Approach 3: analysis using last EQ5D value when reported recovery for missing cases If they had returned to normal

|  | Baseline | 1 month | 4 months | 12 months |
| --- | --- | --- | --- | --- |
| Mean | 0.91 | 0.61 | 0.78 | 0.81 |
| SE (mean) | 0.01 | 0.01 | 0.01 | 0.01 |
| SD | 0.21 | 0.33 | 0.28 | 0.27 |
| Median | 1.00 | 0.69 | 0.80 | 0.85 |
| IQR | (0.85 to 1.00) | (0.36 to 0.80) | (0.69 to 1.00) | (0.71 to 1.00) |
| Count | 1481 | 863 | 709 | 653 |

Approach 4: analysis using multiple imputation of EQ-5D values in all non-responders (including recovered cases), excluding single case of poisoning

|  | Baseline | 1 month | 4 months | 12 months |
| --- | --- | --- | --- | --- |
| Mean | 0.91 | 0.63 | 0.79 | 0.81 |
| SE (mean) | 0.01 | 0.01 | 0.02 | 0.01 |
| SD | 0.21 | 0.33 | 0.27 | 0.26 |
| Median | 1.00 | 0.69 | 0.81 | 0.85 |
| IQR | (0.85 to 1.00) | (0.43 to 0.88) | (0.69 to 1.00) | (0.73 to 1.00) |
| Count (total number of values, 10 for each of 1516 people) | 15160 | 15160 | 15160 | 15160 |
